# The complexity of DNA damage by radiation follows a Gamma distribution: insights from the Microdosimetric Gamma Model

**DOI:** 10.3389/fonc.2023.1196502

**Published:** 2023-06-16

**Authors:** Alejandro Bertolet, Ibrahim Chamseddine, Harald Paganetti, Jan Schuemann

**Affiliations:** Department of Radiation Oncology, Massachusetts General Hospital and Harvard Medical School, Boston, MA, United States

**Keywords:** DNA damage, TOPAS-nBio, microdosimetry, MGM, particle therapy

## Abstract

**Introduction:**

DNA damage is the main predictor of response to radiation therapy for cancer. Its Q8 quantification and characterization are paramount for treatment optimization, particularly in advanced modalities such as proton and alpha-targeted therapy.

**Methods:**

We present a novel approach called the Microdosimetric Gamma Model (MGM) to address this important issue. The MGM uses the theory of microdosimetry, specifically the mean energy imparted to small sites, as a predictor of DNA damage properties. MGM provides the number of DNA damage sites and their complexity, which were determined using Monte Carlo simulations with the TOPAS-nBio toolkit for monoenergetic protons and alpha particles. Complexity was used together with a illustrative and simplistic repair model to depict the differences between high and low LET radiations.

**Results:**

DNA damage complexity distributions were were found to follow a Gamma distribution for all monoenergetic particles studied. The MGM functions allowed to predict number of DNA damage sites and their complexity for particles not simulated with microdosimetric measurements (yF) in the range of those studied.

**Discussion:**

Compared to current methods, MGM allows for the characterization of DNA damage induced by beams composed of multi-energy components distributed over any time configuration and spatial distribution. The output can be plugged into ad hoc repair models that can predict cell killing, protein recruitment at repair sites, chromosome aberrations, and other biological effects, as opposed to current models solely focusing on cell survival. These features are particularly important in targeted alpha-therapy, for which biological effects remain largely uncertain. The MGM provides a flexible framework to study the energy, time, and spatial aspects of ionizing radiation and offers an excellent tool for studying and optimizing the biological effects of these radiotherapy modalities.

## Introduction

Ionizing radiation is a cytotoxic agent used to treat cancer by damaging or destroying cancer cells through physical and chemical interactions. However, radiation can also have unintended effects on normal cells, making it important to carefully balance the exposure of cancer cells and normal cells to apply radiation-based therapies effectively. Multiple factors influence the effect of irradiation on biological systems, and balancing treatment outcomes (tumor control versus normal tissue toxicity) can be challenging.

The absorbed dose, or the average amount of energy deposited per unit mass by radiation, is being used to quantify radiation exposure. Its correlation with outcomes has been shown in a wealth of preclinical and clinical data (1–5). However, it is also well known that the same absorbed dose can lead to different effects depending on the timing of exposure, the microscopic concentration of ionizations in biologically sensitive structures, or the inherent radiosensitivity of different biological systems in different states (6–11).

Radiation therapy using beams of protons, light ions, and heavy particles is becoming more common for cancer treatment due to their ability to target tumors with no or minimal dose beyond the beam’s Bragg peak (12–15)—the region where the radiation beams stop and lose the maximum amount of their energy per path length. These beams also concentrate energy deposition in specific local areas. This differs from X-ray beams, which distribute energy more uniformly. The heavier and slower the particle, the more pronounced this pattern of local energy deposition becomes. Linear energy transfer (LET) is often used to quantify this effect. In addition to ion-based external radiotherapy, targeted internal alpha therapy (TAT) is a novel therapeutic approach utilizing elevated LET (16, 17). In each of these modalities, relative biological effectiveness (RBE) calculations are required to compare the administered doses to those of a reference radiation modality, typically conventional Co-60-based external radiotherapy. This step is necessary to benefit from the rich outcome data from traditional X-ray-based therapies over the past decades. Accurate models of the elementary physics interactions between radiation and tissues are used in Monte Carlo track structure (MCTS) simulations (18–20), which can compare the differences between radiation types and their LETs. However, due to the great computational demands, such simulations are impractical at the clinical scale. As a result, higher-level models of the small-scale nature of radiation–tissue interactions are necessary for clinical predictions of the effects of radiation.

Microdosimetry, which studies the distribution of energy deposited by ionizing radiation at small scales, such as structures at the cell and cell nucleus scales, can provide insight into the radiobiological effects of different types of radiation (21, 22). We have previously shown that microdosimetric quantities can be analytically calculated (23, 24) and that they are correlated with the number and complexity of DNA damage induced by protons and alpha particles (25). Microdosimetry has previously been used as input for biophysics models, particularly the Microdosimetric Kinetic Model (MKM) (26) and its variations (27–31). However, these models rely on statistical assumptions about the distribution of ionization around radiation tracks. While they provide valuable insights, it is important to note that they do not explicitly account for damage induced by reactive oxygen species (ROS) at the chemical stage. The diffusive distance of ROS, particularly hydroxyl radicals, can be in the order of several base pairs (32) and their effects could be captured to some extent by microdosimetric or nanodosimetric models. However, their inclusion is not as straightforward due to variability in scavenging capacity in different cellular environments and at different stages of the cell cycle. Moreover, it is believed that high linear energy transfer (LET) radiation induces less indirect damage (33, 34), although the extent of this reduction can vary. The decrease in indirect damage with high LET radiation is generally attributed to the enhanced rate of recombination (OH + ·OH → H_2_O_2_) due to the proximity of the initially generated species. Our recent study provides quantitative data on these effects using an *in silico* model (35). In addition, MKM and its modifications incorporate radiobiology-related parameters directly as modifiers of the effects described in physical quantities. For example, the modified MKM proposed by Kase et al. (36) uses saturation-corrected parameters to change experimental and theoretical microdosimetric quantities, such as specific energy. Still, these parameters encompass many processes together in a single fit, as biological processes beyond physical processes are at the core of these phenomena. Additionally, their determination is challenging and usually done based on the best fit to the experimental data. These limitations challenge the generality of the model and its application to different biological or physical settings, such as repair-related gene inactivation or mutation or low-dose-rate irradiations in which DNA damage induction and repair coincide.

**Figure 1:**
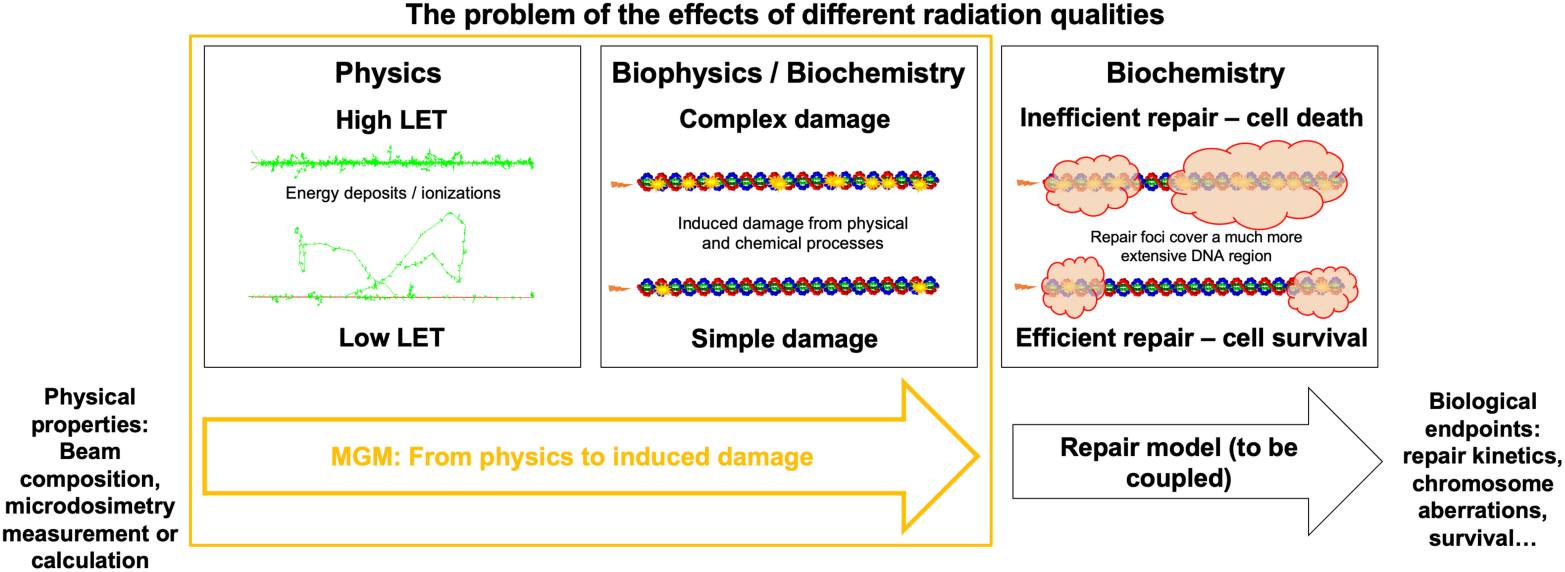
Accelerating DNA damage evaluation using MGM. Representation of the radiation biology problem in the context of different radiation types: from the physical properties of a track, different DNA damages will be induced, which can be predicted in an analytical (i.e., fast) way by the MGM. Repair models are required to determine biological endpoints for different cells after DNA damage is induced.

This study overcomes the current limitations of microdosimetric approaches toward relative biological effects across radiation types. We used TOPAS-nBio (35, 37, 38) to generate radiation-induced damage data for monoenergetic 250 keV X-rays and also for monoenergetic protons and alpha particles of different LET. We examined the relationship between these results and microdosimetric quantities. We investigated the viability of microdosimetry as a proxy for the yield and complexity of damage induced by a single track and presented a new model based on microdosimetry to accurately account for damage induction in detail rather than relying on higher-level experimental results. We defined a complexity metric for the damage sites calculated with TOPAS-nBio and assessed whether this metric follows a Gamma distribution. Our resulting model is called the Microdosimetric Gamma Model (MGM) and predicts the number of DNA damage sites and their complexity. The MGM can be directly connected to system-specific repair models, potentially separating events at different time and space scales. **Figure 1** illustrates the process of translating different radiation beams into biological effects and the role that the MGM plays.

## Methods and materials

### Simulations with TOPAS-nBio

We used MCTS simulations and the TOPAS-nBio (v2.0) toolkit to characterize the DNA damage induced by different radiation types. TOPAS-nBio simulates radiation tracks and a nucleus model containing the whole human genome, allowing the energy at each nucleotide to be computed (38). It also considers the chemical stage of the interaction between radiation and tissue, explicitly simulating the diffusion and reactions between ROS and the induction of ROS-mediated damage to the DNA nucleotides (39). The detailed nature of the MCTS simulations allows us to study the intrinsic differences in DNA damage induction across radiation types and LET.

DNA nucleotides are composed of two moieties: a base and a sugar-phosphate backbone. These volumes do not have an internal structure in TOPAS-nBio but rather are represented by an arbitrary space where energy may accumulate to alter the molecular stability of DNA. In this work, bases were represented as oblate spheroids with semi-major axes of 0.328 nm and semi-minor axes of 0.185 nm, and backbones were represented as 0.271 nm-radius spheres (40). These settings must be consistent with the criteria selected to determine when damage occurs in each moiety. The criteria used to calculate the induced direct, quasi-direct, and indirect DNA damage (35) could be retrieved independently.

In TOPAS-nBio, a generic human cell nucleus model in the G1 cell cycle phase is created as follows: nucleotides are linked in a double-helix shape and coiled around histones, represented as cylinders, to form a nucleosome of 5.7 nm in length. Nucleosomes are then arranged helically to form 120-nm straight chromatin fibers. These chromatin fibers are arranged in a 3D pattern following the Hilbert curve to fill cubes of 0.3 μm on each side containing 0.42 Mbp of DNA. These cubes are repeated three-dimensionally to fill the space within a sphere of 9.65 μm in diameter, representing the cell nucleus. Chromosomes are represented as groups of these cubes. For more details, see Zhu et al. (38).

We simulated the tracks of various radiation types traversing a cell nucleus uniformly across its projection. Monoenergetic 250-keV X-rays; protons with initial energies of 1, 2, 5, 10, 20, and 100 MeV; and α-particles with initial energies of 2, 4, 6, 8, 10, 15, 20, 30, and 50 MeV were simulated in liquid water, adding the corresponding margins to ensure electronic equilibrium in the cell nucleus. The Geant4-DNA ‘option 2’ physics list (41, 42) was employed to determine the cross sections of electromagnetic processes. After simulating the physical stage, water dissociation was simulated, and ROS species were generated. ROS were allowed to diffuse and react using the latest TOPAS-nBio reaction rate tables (43). The chemical stage was simulated step-by-step, starting at 1 ps and stopping at 1 ns after irradiation in 0.5 ps steps, following the methodology of our previous study (35). The output of these simulations was DNA damage as per the Standard DNA Damage (SDD) specification (44). Damage scoring options used in TOPAS-nBio were selected as specified in **Table 1**. In this standard, the damage is reported by ten base pair-length damage sites, specifying the number and origin of lesions contained.

**Table 1:** Parameters used to determine when damages occur.

Parameter	Value
Energy deposited threshold to consider direct damage (for both moieties)	11.75 eV
Probability of charge transfer from hydration shell to backbones	0.33
Probability of charge transfer from hydration shell to bases	0.67
Probability of scavenging ROS in a step within backbones	0.25
Probability of scavenging ROS in a step within bases	1.0
Probability for a ROS to induce indirect damage in a step to backbones	0.55
Probability for a ROS to induce indirect damage in a step to bases	1.0

Results were stored on a track-by-track basis and post-processed to read and analyze SDD files up to a particle fluence equivalent to 6 Gy. To compute the yield of damage induced per Gy and account for the stochasticity of radiation-matter interactions, we bootstrapped a subset of tracks reaching various doses 40 times and calculated the mean values and variances. We allowed damage at the same sites from independent tracks to accumulate. We defined the complexity of a damaged site as the number of total bases and backbones damaged, and we computed distributions of damage complexity including only those containing at least one double-strand break (DSB). A formal definition of complexity score in this work is provided in the next section.

### Microdosimetry calculations and the core of the MGM

Microdosimetric spectra for each of the radiation types employed in this work were calculated on a spherical site made of liquid water, as detailed elsewhere (24, 45, 46). The spherical site was made to match the nucleus size in the TOPAS-nBio simulations (9.65 μm diameter). Lineal energy ( y ) is the total energy deposited within the site in a single event divided by the site-corresponding mean chord length, or two-thirds of the site diameter. As the MGM provides a measure of DNA damage by track, the track- or frequency-weighted average lineal energy, $y_{F}$ , was selected as the independent variable to predict the DNA damage characteristics of each spectral component of a beam. In this work, $y_{F}$ was calculated for 1 μm diameter sites. This choice reflects common practice in microdosimetry and avoids recalculating microdosimetric quantities for each specific case or nucleus volume.

We assessed how different damage events in the DNA relate to the microdosimetric properties of each radiation type. Direct damage by a track can be considered proportional to the energy deposited by that track and, in turn, proportional to $y_{F}$ . However, indirect damage after water radiolysis saturates when the ionization density increases due to the recombination of ROS. This dependence was modeled as $N_{I}\left(y_{F}\right)=N_{max}\left[1-\exp\left(-a\;y_{F}\right)\right]$ , where $N_{I}$ is the number of damages induced by the indirect effect, and $N_{max}$ and a are free parameters obtained by least square fits. These two dependencies for direct and indirect damage were assumed concerning the total number of strand breaks (SB), base damages (BD), and damage sites (DS) per ionizing track. A DS was defined as a block of damage occurring in 10 consecutive DNA base pairs, in the same way as specified in the SDD output (44). The total number of DS defined this way was assumed to follow the same dependence on $y_{F}$ as SBs or BDs. However, arguably, the total number of DS is not relevant from a radiobiological point of view since simple SB or BD typically do not lead to severe consequences for the cell. Therefore, we also computed the number of DSs containing at least one DSB. The number of DSs with at least one DSB induced per ionizing track, was assumed to follow a linear-quadratic dependence to $y_{F}$ , as ionization positions are strongly correlated.

When determining the fate of a cell exposed to ionizing radiation, not only the number of DS with DSBs is important, but also how complex the induced damage is. Multiple breaks concentrated in a DS have been shown to be more challenging to repair (47, 48). In this work, we defined a metric to characterize the complexity of the DS as the total number of strand breaks and the number of base damages contained in the site, provided that at least one DSB is present. Therefore, simple DSBs have a complexity score of 2, and DSs without DSBs do not have any complexity score assigned and are not part of the complexity distribution. In each exposure, a distribution of complexity across DSs can be found. A way to characterize this is by means of a Gamma distribution function, $f\left(C;y_{F}\right)=\frac{b{\left(y_{F}\right)}^{a\left(y_{F}\right)}}{\text{Γ}\left(a\left(y_{F}\right)\right)}C^{a\left(y_{F}\right)-1}\exp\left(-b\left(y_{F}\right)\;C\right)$ . The Gamma distribution uses two parameters, $a\left(y_{F}\right)\text{ and }b\left(y_{F}\right)$ . The first, a , represents how complex the damage is expected to be and how spread out the distribution is. The second, b , controls the shape of the probability density function at high complexity. These parameters change with different types of radiation. To figure out how they change, we used second-order polynomials to model the relationship between a , b , and $y_{F}$ across the protons and alpha particles simulated.

### Repair modeling and biological endpoints

To illustrate how the induced DNA damage can be used as an input to calculate biological endpoints, we used a simplistic repair model that assigns a given probability for a DS to become irreparable and, ultimately, lethal as a function of its complexity. We modeled this using a sigmoid function of the complexity C , i.e., $p_{dam\to lethal}\left(C\right)=1/(1+\exp\left(-d\left(C-C_{0.5}\right)\right)$ , where d is a cell-dependent parameter that controls how efficient DNA damage repair is; and $C_{0.5}$ is a cell-dependent parameter that specifies at what complexity the probability of becoming lethal is 50%. After N DSs, the resulting probability of cell death is $P_{lethal}=1-{\text{Π}}^{N}\left(1-p_{dam\to lethal}\right)$ . In order to show realistic results in two cases of interest, we utilized the data published by Hill et al. (49) and Jones et al. (50), in which repair efficient V79-4 and repair impaired irs1 hamster cell lines were exposed to 250-kVp X-ray and 3.26 MeV alpha particles. We fitted our $p_{dam\to lethal}\left(C\right)$ model to reproduce the results of these experiments and calculated the cell survival curves. We then calculated the required doses to obtain 90%, 50%, and 10% survival as a function of $y_{D}$ using these repair models. RBE was obtained as the ratio of doses with X-rays and any radiation to achieve the same survival level. RBE in the limit of very low dose (RBE min) was also calculated as the fraction of the α parameter determined upon fits of the model to the clonogenic survival predictions.

### Model validation and damage generation

To validate the predictive ability of our functions, we simulated alpha particles of 3 MeV and 5 MeV in TOPAS-nBio. We compared the number of DSs and their complexity with the calculated damage complexity using MGM. The differences between simulated and predicted distributions of damage complexity were measured using the root mean square error (RMSE). We used the number of DSs per track and the Gamma distribution of complexity to generate spatial distributions of DSs for a given radiation dose, distributing the sites in straight lines as particles usually do. The source code for the MGM can be found in a public repository on Github: https://github.com/mghro/mgm.

## Results

### Damage induced by radiation types

**Figure 2:**
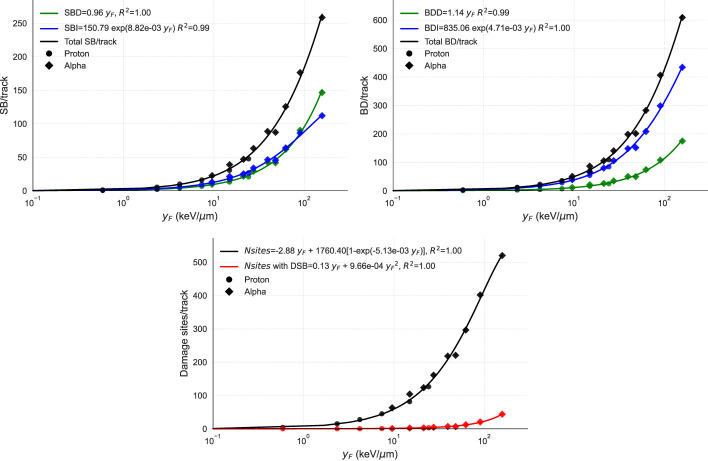
Points show the DNA damage induction as a function of $y_{F}$ from TOPAS-nBio simulations. Solid lines show the performance of MGM, which uses the relations given in the legends. The three panels display the relationship between $y_{F}$ (in logarithmic scale) and (top left) the number of strand breaks per track, broken down into direct (SBD), indirect (SBI), and total (SB); (top right) the number of base damages per track, also specified by direct (BDD), indirect (BDI). and total (BD); and (bottom) the number of damage sites per track with and without double-strand breaks, with fits of the proposed functions. In the top panels, direct, indirect, and total damages are represented in green, blue, and black, respectively. In the bottom panel, total DSs are represented in black and DSs with one DSB in red. TOPAS-nBio results for protons and alpha particles are marked with circles and diamonds, respectively.

We used the MGM model to simulate various radiation types and evaluate their effect on strand breaks, base damages, and DSs per track as a function of $y_{F}$ (**Figure 2**). To validate the results, we simulated the same scenarios on TOPAS-nBio. The assumptions of the MGM for direct and indirect damage showed excellent agreement with the simulated data. The number of DNA DSs exhibited a combination of linear and saturated dependence on $y_{F}$ , while the number of DSs containing at least one DSB displayed a linear-quadratic relationship with $y_{F}$ .

**Figure 3:**
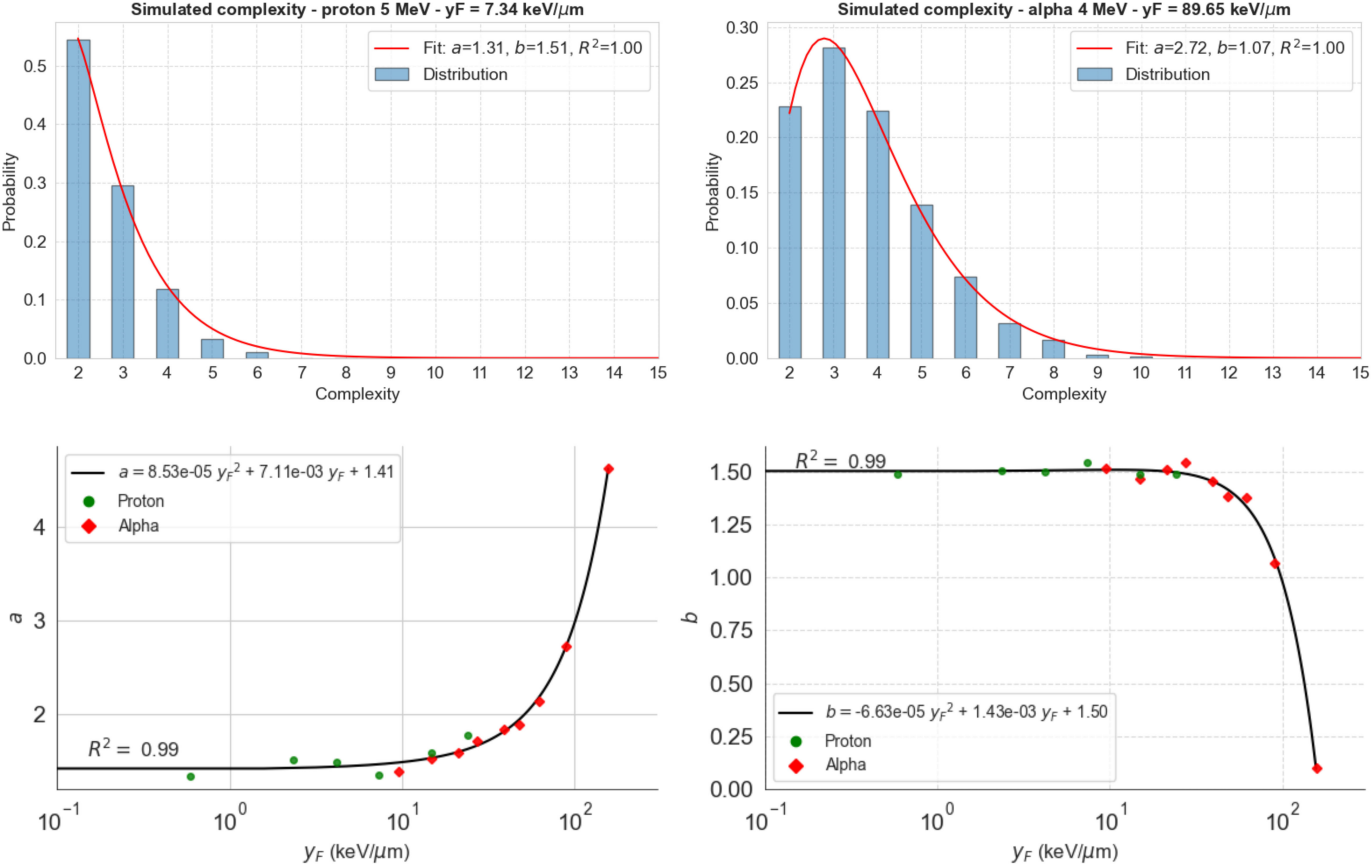
Distribution of complexity for 5-MeV protons (top left) and 4-MeV alpha particles (top right) calculated using TOPAS-nBio. Gamma distribution (red line) fits the data in both cases (R^2^ >0.999). Bottom panels show the dependence of the found parameters on $y_{F}$ (logarithmic scale) and a second-order polynomial fit to calculate the values of these parameters at any $y_{F}$ .

**Figure 4:**
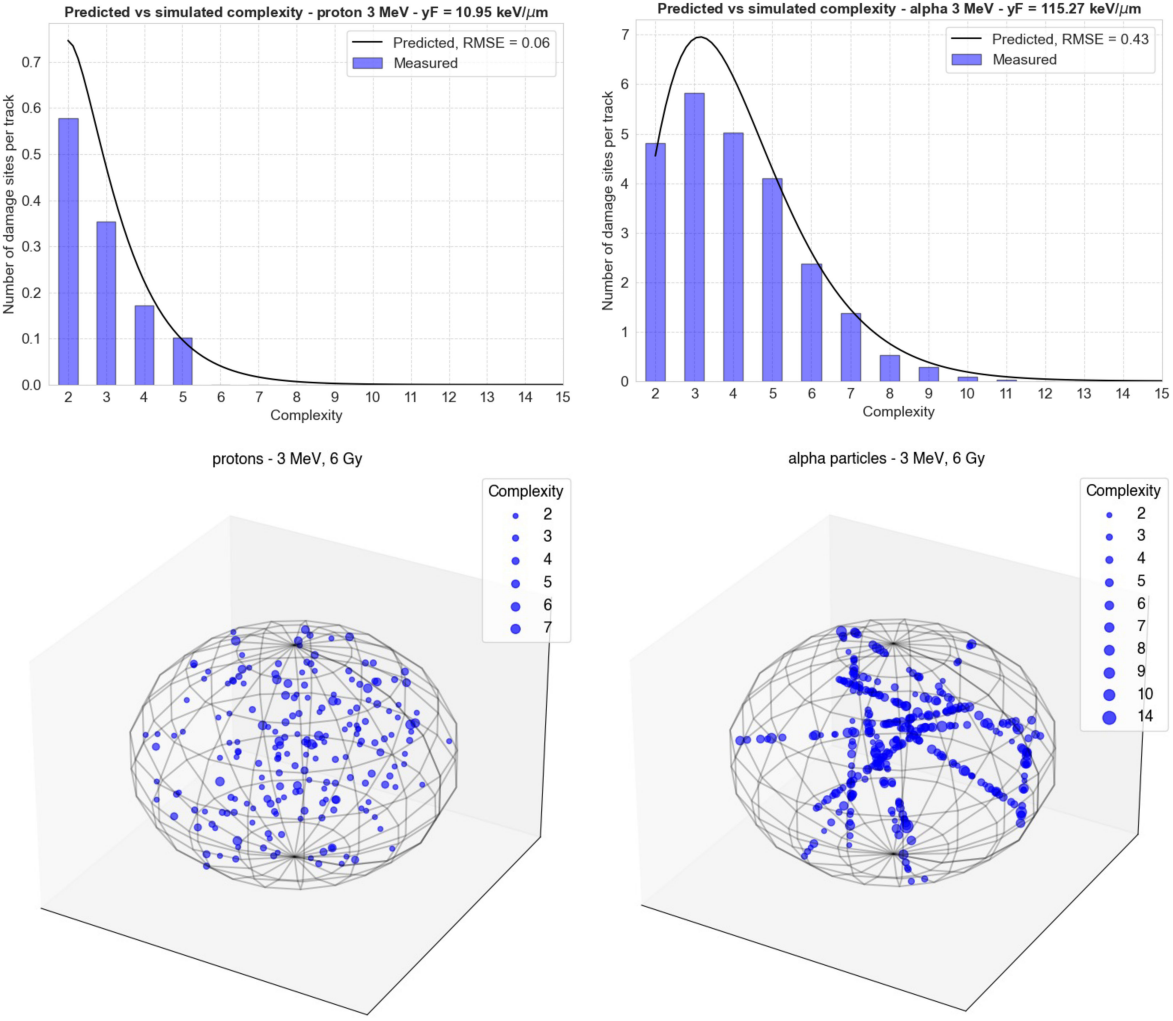
Comparison of the Gamma distributions calculated using the functions from **Figure 2** (black lines) with new simulations performed in TOPAS-nBio (blue bars). The top left panel shows the results for 3-MeV protons ( $y_{F}$ = 10.95 keV/μm) and the right panel shows the results for 3-MeV alpha particles $y_{F}$ = 115.3 keV/μm). The bottom panels show the spatial distribution of damage sites generated by MGM in each case in a spherical cell nucleus. The size of each point increases with the complexity of the damage.

We then used the model to study the distribution of complexity for 5-MeV protons and 4-MeV alpha particles as examples of how the Gamma distribution fits the calculated complexity data (**Figure 3**). Gamma distributions reproduced extremely well the observed DS complexities for both radiation particles and all energies analyzed. The Gamma-specific parameters vary with $y_{F}$ (bottom panels), which can be utilized to predict the Gamma-complexity distribution of any track after determining its $y_{F}$ value. The **Supplementary Material** includes the rest of the Gamma distributions fitted to our simulation data. We calculated the Gamma distributions for each function using TOPAS-nBio (**Figure 4**).

In **Figure 4**, the comparison of the Gamma distributions calculated using the functions from **Figure 3** with new simulations performed in TOPAS-nBio is shown. The left panel displays the results for 3 MeV protons, with $y_{F}$ = 10.95 keV/μm, and the right panel shows the results for 3 MeV alpha particles, with $y_{F}$ = 115.3 keV/μm. This comparison allows for a validation of the accuracy of the functions determined in **Figure 3**, and the results demonstrate a relatively good agreement between the calculated distributions and the new simulations, although low-complexity DSs were underestimated.

### Repair probability and cell survival

**Figure 5:**
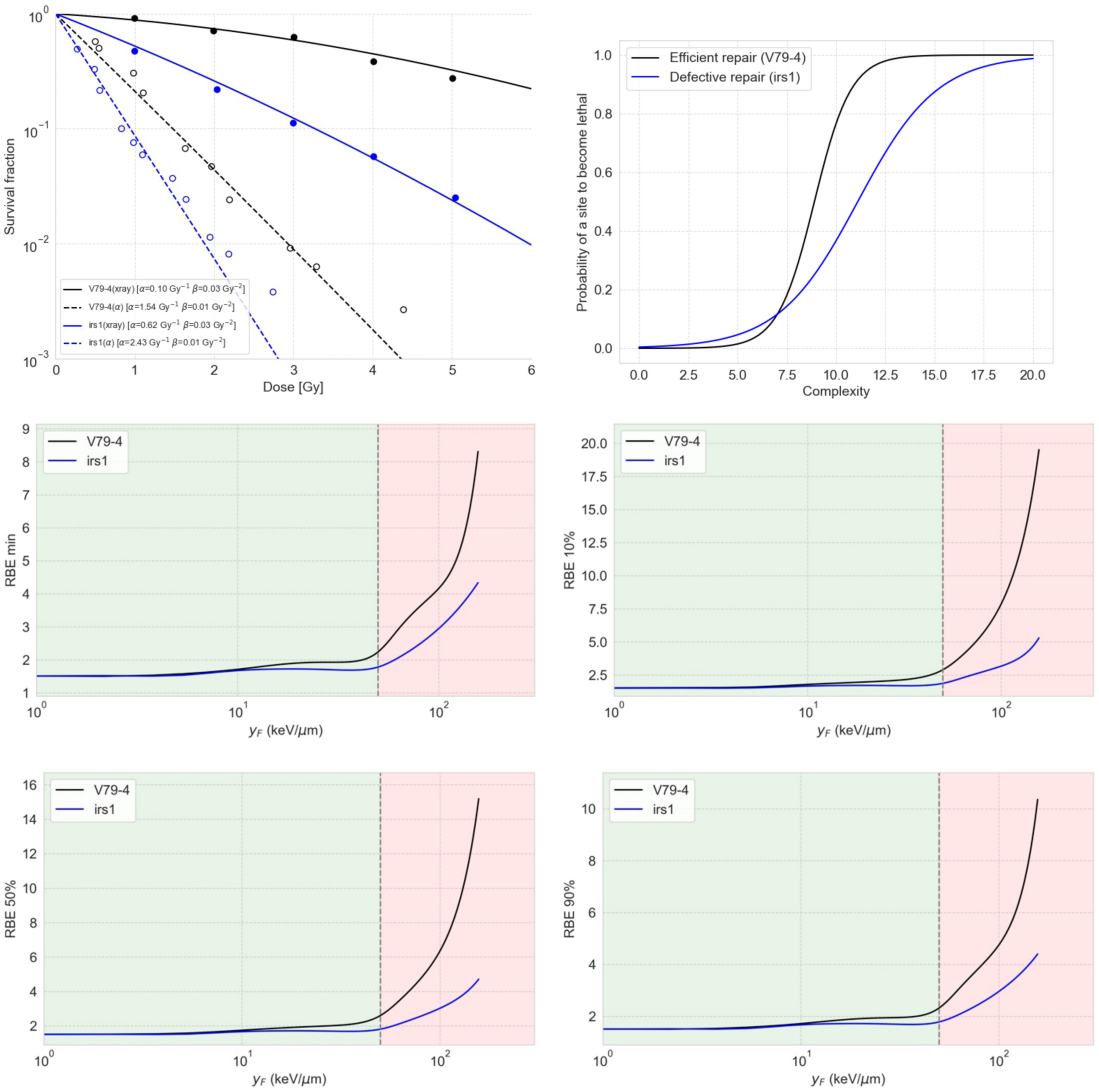
The top left panel shows the predicted survival curves for V79-4 and irs1 cell lines using the sigmoid repair models shown on the top right panel. The middle and bottom panels show RBE as a function of $y_{F}$ , estimated using the damage complexity distribution by the MGM together with a sigmoid repair model, for V79-4 and irs1 cell lines. The four panels show the RBE values for different levels of cell survival, including the minimum RBE (middle left), the RBE at 90% survival (middle right), the RBE at 50% survival (bottom left), and the RBE at 10% (bottom right).

We predicted survival fraction curves for two cases, the V79-4 cell line (efficient repair for simple damages) and irs1 (deficient repair), using a sigmoid repair model connected to the damage predicted by the MGM (**Figure 5**, top left). The irs1 cell line is isolated from mutagenized cultures of V79-4 and is defective in RAD51-like genes and XRCC2, which has been shown to have a reduced ability to use homologous recombination for DNA repair (49). We found the sigmoid functions (**Figure 5**, top right) that best reproduced the experimental data for 250-kV X-rays and 3.26-MeV alpha particles published elsewhere (49, 50). It is important to notice that our simulations for induced damage from X-rays referred to a monoenergetic 250 keV spectrum and not a 1.5 mm Al-filtered 250 kV spectrum. In this sense, using a spectrum instead of pristine radiation types led to a loss of the Gamma-like behavior of the complexity distribution. Noticeably, representing a defective repair for low-complex damage required a more spread-out sigmoid to reproduce the data, which generates the strange notion of the curve of defective repair falling below the one for efficient repair for high complexity scores.

This observation in **Figure 5** arises from the symmetry inherent in the sigmoid model for repair. It is crucial to note that this model is not designed to realistically represent repair processes but to serve as a conceptual tool for interpreting complexity distributions. Most of the damage sites appear to have complexity equal to or less than 7, within which the efficient repair curve dips below the defective repair curve for lethality.

We also calculated the dependence of RBE on $y_{F}\;for$ different cell survival fractions, as shown in **Figure 5**, for both cell lines using the combination of MGM and the correspondent sigmoid repair model. Our results indicated that cells with deficient repair are less sensitive to RBE compared to cells with proficient repair. This means that proficient-repair cells are more susceptible to the enhanced effects of complex DNA damage, while cells with deficient repair exhibit higher baseline effects, making them more prone to death from simple DNA DSs.

## Discussion

The MGM concept presented in this work provides a track-based approach to predicting DNA damage distributions in radiation therapy scenarios, including proton therapy, targeted therapy, and helium therapy. This approach combines different track types and energies, spatial distributions, and time patterns for dose deposition. MGM provides as an output the distribution of DNA damage and its complexity, which happen during the initial chemical stage of the interaction between radiation and living systems. This study reveals two important findings that can improve our understanding of radiation-induced damage. First, we found that the distribution of damage complexity induced by radiation follows a Gamma distribution, with specific trends in the parameters as the quality of radiation increases. This means that we can predict the complexity of damage for a given radiation using mathematical functions rather than relying on computationally expensive and time-consuming simulations, opening the door for large, longitudinal studies comparing radiation types and their biological effects. Second, our model enables us to capture the differences among different cell lines in a much more detailed way than other approaches, such as MKM, which rely directly on higher-scale functions fitted to experimental results for clonogenic survival. By predicting the number and complexity of damage induced by radiation, our model opens the door to many subsequent biologically driven processes that happen in response to radiation. While our model needs to be coupled with other detailed models to figure out the biological response to radiation, it provides an important framework for understanding how different types of radiation can induce damage, which is crucial for developing effective radiation therapies. In practice, the MGM can be applied to multi-energetic beams by calculating the average values for $N_{sites}$ using the averages $\bar{y_{F}}$ and $\bar{y_{F}^{2}}$ (or the mean-dose lineal energy, $y_{D}\equiv \bar{y_{F}^{2}}/\bar{y_{F}}$ as this can be calculated analytically (23, 24) or measured experimentally) across the beam spectrum. An implementation of this technique is available at github.com/mghro/mgm.

Specifically, the MGM can be used to predict the damage induced by, at least, any radiation with $y_{F}$ between the limits shown in this paper, i.e., around 2 keV/μm (100-MeV protons) and 200 keV/μm (2-MeV alpha particles). We compared these predictions for 3-MeV protons and 3-MeV alpha particles, demonstrating a good agreement between the calculated distributions and new detailed simulations, although low-complexity DSs were slightly underestimated. Of note, we chose predictions in particularly challenging situations, as these radiations are found in the zone of rapid change for the a and b parameters. Their impact on survival results depends on the repair model connected to the DNA damage induction step.

As mentioned, one of the key advantages of the MGM over other track-structure-based models, such as the MKM or the local effect (LEM), is its ability to capture indirectly induced damage, as the interaction of ROS with DNA is explicitly simulated in TOPAS-nBio and thus captured by the MGM. Additionally, the output provided by the MGM can serve as the basis for other measurable biological endpoints, such as protein recruitment dynamics for repair foci or the number of chromosome aberrations. In this work, we illustrated how to use the output of the MGM to calculate the survival fraction and the RBE for this endpoint for two cell lines with enabled and impaired repair abilities by connecting the predicted damage complexity to a sigmoid-based repair model specifically designed for our scenario. For example, an unrealistic dip around 20 keV/μm can be observed for the RBE defective repair cells in **Figure 5**, which is an artifact coming from the unstable survival curves produced by the overly simplistic sigmoid repair model. Therefore, the results shown in **Figure 5** must be considered qualitatively, including the interpretation of high or low sensitivity to RBE for defective or efficient repair cells. More mechanistic repair models can be linked to the MGM. One of the future directions after this work is the development of more nuanced repair models that consider the distributions of damage complexity.

The shown relationship between $y_{F}$ and the number and complexity of DNA DSs is specific to the nucleus model utilized in TOPAS-nBio, which assumes a uniformly distributed genome in the G1 phase. We chose $y_{F}$ as the variable to represent radiation quality due to its robustness as opposed to $z_{F}$ , i.e., the mean specific energy, which depends on the size of the DNA container. However, for basic geometries such as spheres, $y_{F}\text{ and }z_{F}$ are simply related by $y_{F}=z_{F}\;\bar{l}/m$ , where m is the sphere’s mass and $\bar{l}$ its mean chord length. As a result, $z_{F}$ could be used as the independent variable to predict the number of DSs, which can be scaled to account for the DNA contained in other scenarios. Additionally, as previously shown (25), adjusting the size of the microdosimetric site allows predicting the number of DSB and complex damages for different chromatin folding by using $z_{F}$ while keeping the same functional relationships. This would allow the characterization of different DNA compaction and arrangement (i.e., different cycle stages) by transforming $y_{F}$ into $z_{F}$ values based on the specific operational size of the microdosimetric site in each scenario. Determining this operational size for $z_{F}$ and its variability across biologically realistic situations will require additional simulations with DNA folded in different arrangements. Provided these values are known, the track-by-track feature of the MGM will allow us to explicitly consider variations in the amount and complexity of the DSs at different cycle stages, which is, in turn, combinable with cell cycle-dependent repair models.

The MGM functions are subject to some limitations. TOPAS-nBio, when used as indicated in our previous work (35), reproduces the yield of DSB reported in the literature at low LET radiations. However, further benchmarking for damage complexity at high LET radiations would be desirable. Secondly, the relations found in the MGM are valid if the so-called short track condition in microdosimetry is maintained, meaning that the particle loses approximately the same amount of energy per unit length within the microdosimetric site. For very low-energy particles, for example, 500-keV protons or 1-MeV alpha particles, this assumption does not hold. It leads to skewed spatial distributions of ionizations within the microdosimetric site, which can impact the amount and type of DNA damage. Additionally, the current version of the MGM is limited to protons and alpha particles, and its applicability to heavier ions is unclear. Future simulations with TOPAS-nBio will be conducted to incorporate, at least, carbon ion therapy into the MGM scope. The MGM helps provide only cell nucleus-based effects. It cannot capture other phenomena related to extra-nuclear effects within a cell, tissue, or tumor microenvironment or immune responses.

Further considerations regarding the generalizability of our nucleus model in TOPAS-nBio are pertinent. First, we consider no differences between heterochromatin and euchromatin. However, while heterochromatin areas impede the accessibility of repair molecules and are hence more susceptible to cell-threatening damage, it is not clear that the number and complexity of induced damage will significantly change. Therefore, this effect can be addressed at the repair step by including this dependence in repair models. Second, a spherical nucleus of 9.65 μm in diameter is employed, disregarding effects related to the nucleus shape. A way to overcome this limitation is shown in the bottom panels of **Figure 4**. One could use the MGM apparatus to obtain the number of DSs and DSBs along a straight line traversing our 9.65 μm nucleus and then scale this number according to the actual length of the nucleus in different scenarios, distributing the DSs and their complexity along those lines randomly. Additionally, this work focuses on the induction of individual DSs, but DSs can interact pairwise, leading to genetic rearrangements and chromosome aberrations. Again, this effect should be incorporated into the repair modeling. A possible way to do this would be to simulate the diffusion of the DSs generated in the nucleus and decide whether these potential endpoints occur or not as a function of the distance between DSs. Additionally, by separating components in terms of direct and indirect damage, as shown in **Figure 2**, one could consider a variable concentration of radical scavengers by suppressing the corresponding portion of DSs. This operation, however, is not trivial and will be carefully addressed in future versions of our model. In summary, it is important to note that, to account for cell-specific variable environments, the MGM needs to be coupled with a sophisticated model for the repair stage. Conversely, developing such a repair model becomes easier by means of the MGM if the distributions of DSs and their complexity are readily calculable.

The MGM can be used to predict DNA damage complexity in various cases. Besides its application to proton and helium external radiotherapy, when considering TAT, lack of spatial uniformity and protracted irradiations are paramount. By controlling the amount of damage added by each track through the MGM, one can simulate the repair simultaneously and explicitly consider any dose–time curve, as in radionuclide therapy, physical decay and biological washout affect the timespan in which cells are irradiated. It is also important to notice that considering damages per track as independent is only valid for not too large doses (in which DSs become more complex by the addition of the action of multiple tracks). However, in TAT, doses are temporally spaced, and large cumulative doses are rare. Therefore, MGM provides an optimal platform to characterize the effects of alpha particles in this novel treatment modality.

## Conclusions

This study introduces the Microdosimetric Gamma Model (MGM), an analytical method using microdosimetric quantities as input to predict the number and complexity of DNA damage sites induced by the direct and indirect effects of different radiations in proton therapy and targeted alpha therapy. Based on the accuracy of in *silico* experiments using TOPAS-nBio, our results showed that the number and complexity of DNA damage sites increased with the mean lineal energy ( $y_{F}$ ) in predictable ways. In particular, the Gamma distribution provided a good fit for the complexity of the damage sites. The MGM provides as an output the number and complexity distribution of DNA damage per track, which can be used as input for repair process models, including mixtures of radiation and different spatial and time distributions of ionizing tracks. These MGM characteristics provide a useful tool that offers access to DNA damage distributions, which can be used to optimize treatments delivered in proton therapy and TAT.

## Data availability statement

The raw data supporting the conclusions of this article will be made available by the authors, without undue reservation.

## Author contributions

AB conceived the original idea, conducted simulations, produced results, and wrote the original manuscript. JS contributed to the simulations of DNA damage, reviewed the manuscript, and obtained funding for the project alongside AB and HP. IB produced figures and contributed to the manuscript. All authors contributed to the article and approved the submitted version.
