# Supplementary material for: The complexity of DNA damage by radiation follows a Gamma distribution: insights from the Microdosimetric Gamma Model
Source: Front Oncol. 2023 Jun 16;13:1196502. doi: 10.3389/fonc.2023.1196502 (PMC10313124; doi:10.3389/fonc.2023.1196502)
Supplement: Supplementary file 1 [file DataSheet_1.docx]

**Supplementary material**

| 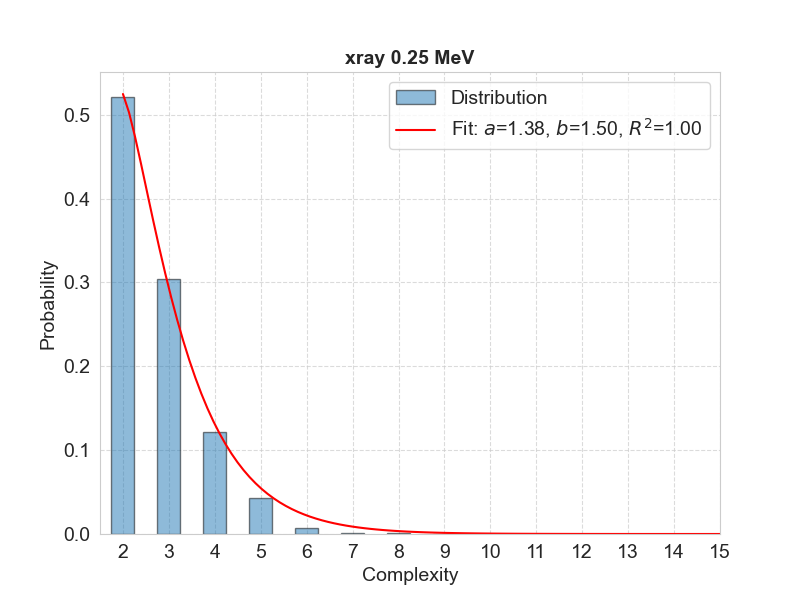 | 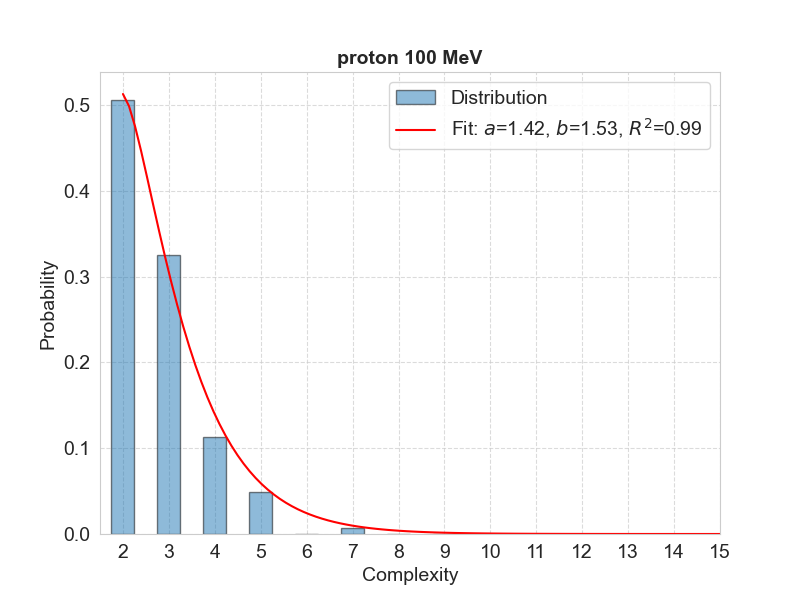 |
| --- | --- |
| 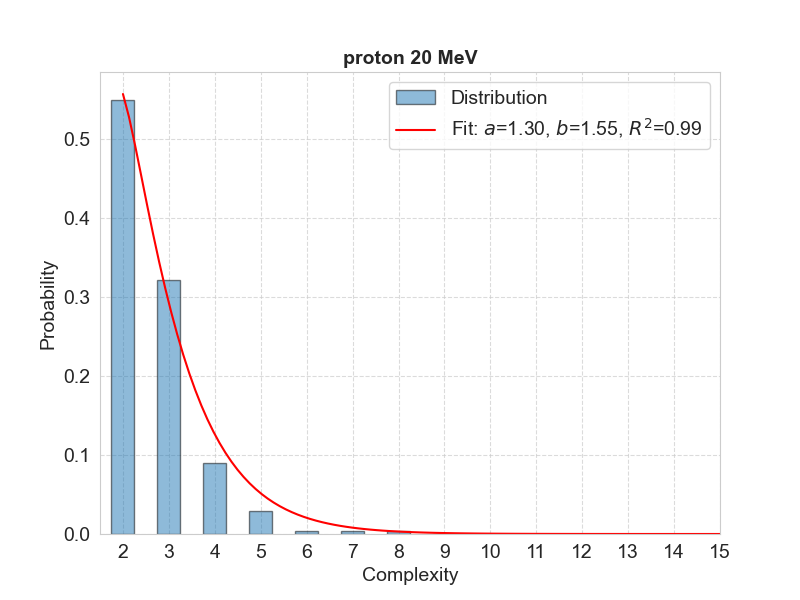 | 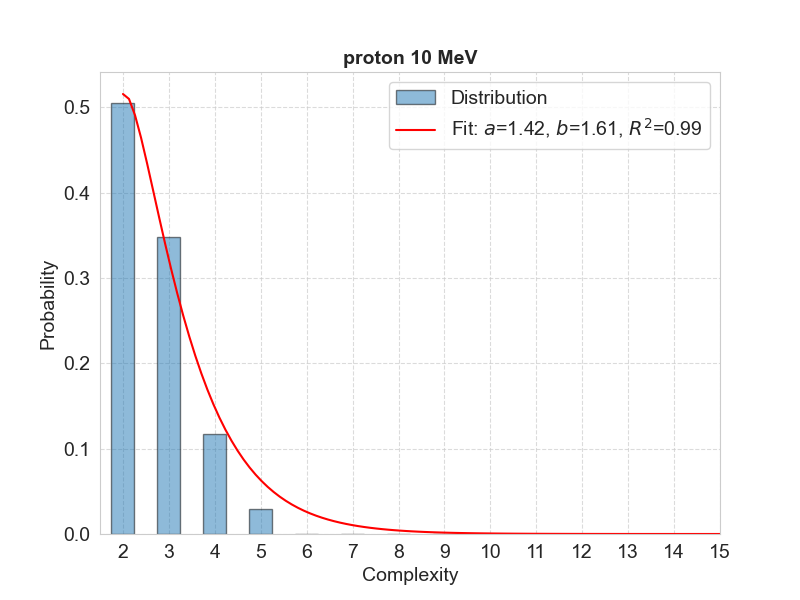 |
| 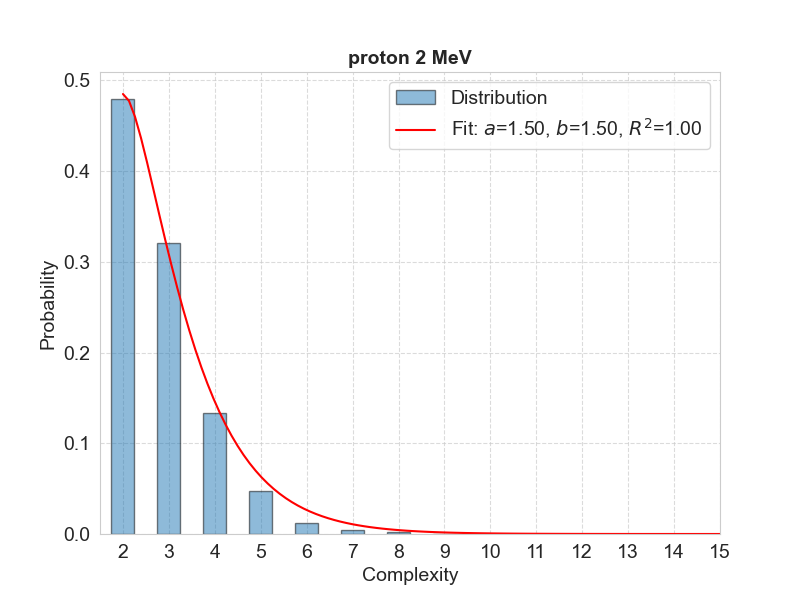 | 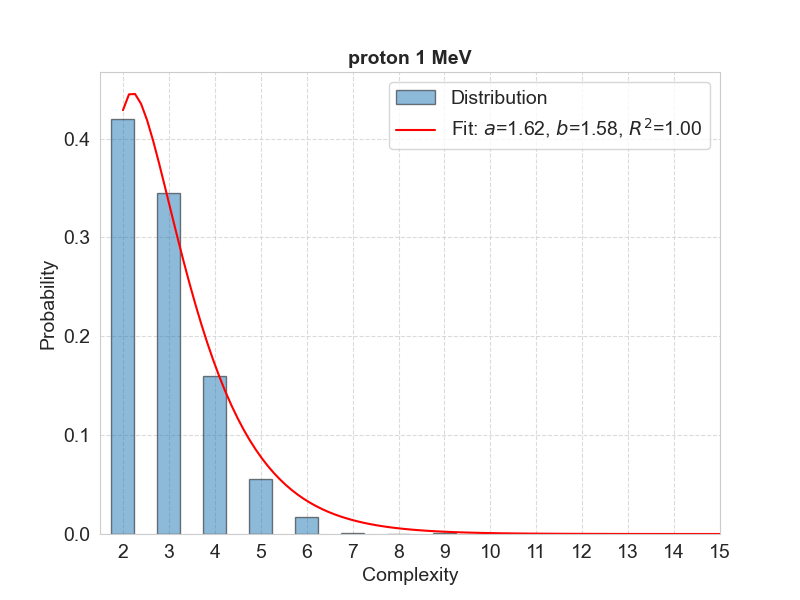 |
| 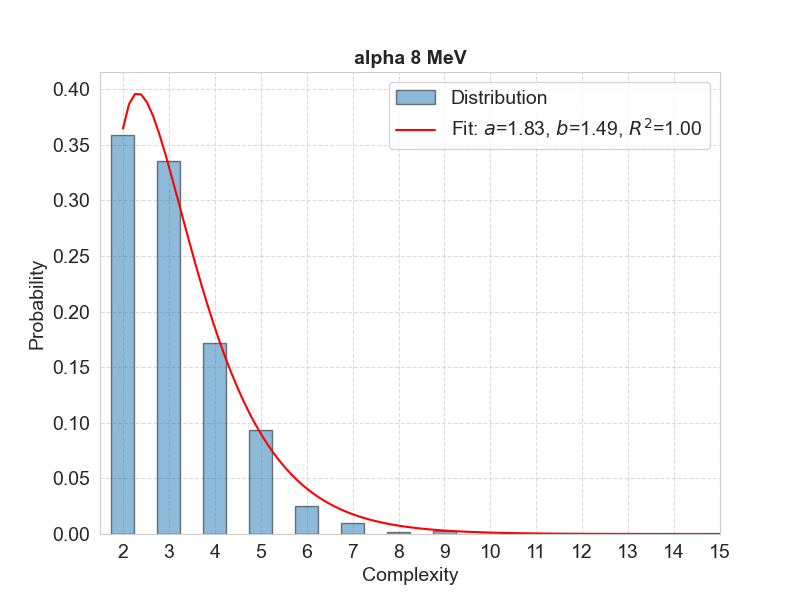 | 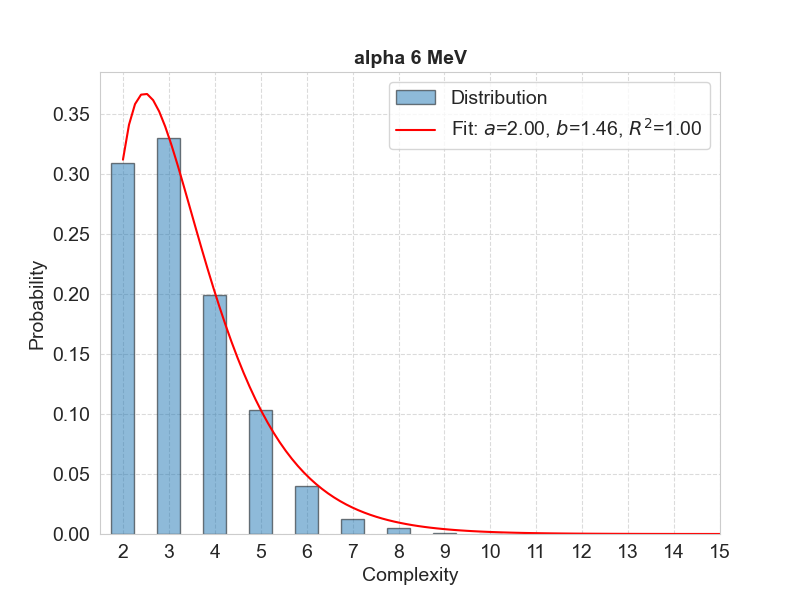 |
| 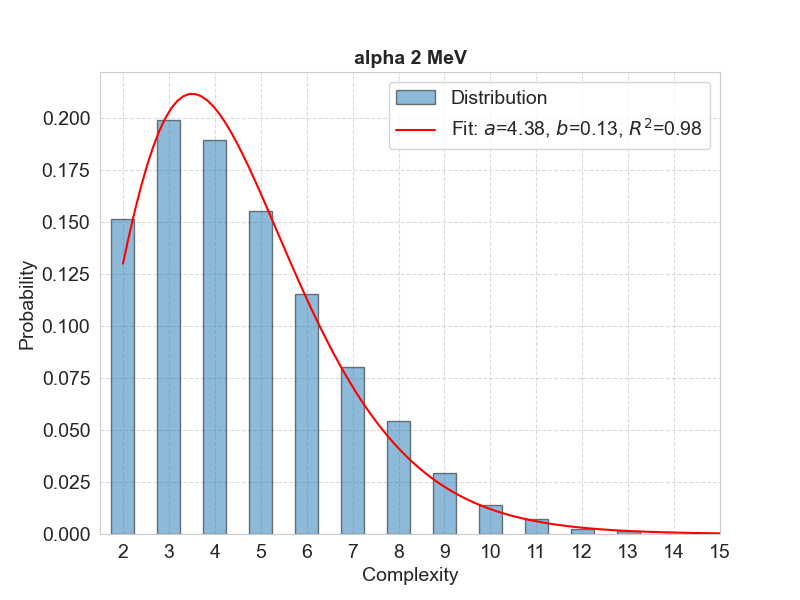 | |
| **Figure S1.** Distributions of complexity for protons and alpha particles simulated with TOPAS-nBio and fits of Gamma distributions to our results. | |
